# The functions and clinical application potential of exosomes derived from mesenchymal stem cells on wound repair: a review of recent research advances

**DOI:** 10.3389/fimmu.2023.1256687

**Published:** 2023-08-25

**Authors:** Xinchi Qin, Jia He, Xiaoxiang Wang, Jingru Wang, Ronghua Yang, Xiaodong Chen

**Affiliations:** ^1^ Zunyi Medical University, Zunyi, China; ^2^ Department of Burn Surgery, The First People’s Hospital of Foshan, Foshan, China; ^3^ Department of Burn Surgery, The First Affiliated Hospital of Sun Yat-Sen University, Guangzhou, China; ^4^ Department of Burn and Plastic Surgery, Guangzhou First People’s Hospital, South China University of Technology, Guangzhou, China

**Keywords:** mesenchymal stem cell-derived exosomes, wound repair, hemostasis, inflammation, immune, proliferation, remodeling

## Abstract

Wound repair is a complex problem for both clinical practitioners and scientific investigators. Conventional approaches to wound repair have been associated with several limitations, including prolonged treatment duration, high treatment expenses, and significant economic and psychological strain on patients. Consequently, there is a pressing demand for more efficacious and secure treatment modalities to enhance the existing treatment landscapes. In the field of wound repair, cell-free therapy, particularly the use of mesenchymal stem cell-derived exosomes (MSC-Exos), has made notable advancements in recent years. Exosomes, which are small lipid bilayer vesicles discharged by MSCs, harbor bioactive constituents such as proteins, lipids, microRNA (miRNA), and messenger RNA (mRNA). These constituents facilitate material transfer and information exchange between the cells, thereby regulating their biological functions. This article presents a comprehensive survey of the function and mechanisms of MSC-Exos in the context of wound healing, emphasizing their beneficial impact on each phase of the process, including the regulation of the immune response, inhibition of inflammation, promotion of angiogenesis, advancement of cell proliferation and migration, and reduction of scar formation.

## 1 Introduction

The skin is the largest multifunctional organ of the human body and serves as a protective barrier against pathogenic invasion as well as chemical and physical assault from the external environment (1–3). Skin trauma resulting from thermal injury or radiation exposure can disrupt structural integrity and internal homeostasis of the skin, leading to wound development. Although the skin possesses inherent self-repair capabilities (4), the wound healing process may occasionally be aberrant, leading to the development of chronic wounds or hyperplastic scars/keloids. Conventional wound management techniques include the use of antibiotics, dressing alterations, negative-pressure wound therapy, debridement, skin grafting and flap transplantation, laser therapy, biological scaffolds, topical administration of specific growth factors, and gene therapy. However, these interventions are associated with potential adverse effects, such as atrophic scarring, abnormal pigmentation, prolonged healing duration, immune rejection, elevated expenses, and increased susceptibility to infection (5).

Recently, stem cell therapy has garnered significant interest because of the pluripotency, self-renewal ability, and capacity of stem cells to stimulate the secretion of regenerative cytokines. Mesenchymal stem cells (MSCs) are pluripotent stem cells derived from the bone marrow and other tissues. As important members of the stem cell family, MSCs can differentiate into various lineages, such as bone, cartilage, and fat. Its positive effects on wound healing have been widely demonstrated. However, in the practical application of stem cell therapy, several limitations have surfaced, including ethical dilemmas, issues pertaining to the source of stem cells, heterogeneity, long-term survival, directional differentiation and proliferation, low migration rates, and chemotaxis rates of stem cells toward the site of injury (6). Furthermore, concerns have been raised regarding abnormal differentiation, immune rejection, teratogenesis, tumorigenesis, and other biosafety issues (7, 8) as well as challenges related to the storage and transportation of stem cells (9).

Interestingly, certain studies have indicated that the biological effects of MSCs can be replicated in conditioned media (10). Subsequent research validated that the modus operandi of MSC therapy encompasses paracrine effects facilitated by MSCs (11–13). Exosomes, which are nanoparticles ranging 30–120nm in diameter enveloped by a bilayer phospholipid membrane, are secreted by biological cells. Initially perceived as mere carriers of cellular waste, numerous studies have established their crucial role in intercellular communication (14). In contrast to stem cell therapy, exosomes offer distinct advantages, including multifaceted biotherapeutic effects, efficient delivery of exogenous cargo, superior biocompatibility, and enhanced safety (15). Furthermore, exosomes exhibit stability and convenience in storage, do not elicit immune system rejection, possess homing ability, and offer ease of dosage regulation (16, 17). Most importantly, extensive studies have shown that mesenchymal stem cell-derived exosomes (MSC-Exos) exert positive effects at all stages of wound healing.

Therefore, this article aimed to review the mechanism of action of the three main types of MSC-Exos in each step of wound healing. Additionally, we discuss the current status of the clinical application of MSC-Exos in wound healing.

## 2 Overview of exosomes

### 2.1 Exosomes and extracellular vesicles

The term “exosome” is commonly employed to refer to all vesicles discharged into the Extracellular milieu by diverse cells; however, it is important to note that exosomes are exclusively a subset of EVs. EVs are membrane vesicles that are released from both the interior and exterior of cells, characterized by a phospholipid bilayer structure and a diameter ranging 50 nm to 5 μm (18). EVs exhibit a high degree of heterogeneity and are capable of being secreted by a diverse range of cell types, including T cells, B cells, dendritic cells, platelets, mast cells, epithelial cells, endothelial cells, neurons, cancer cells, oligodendrocytes, Schwann cells, embryonic cells, and MSCs (19). EVs were first discovered in 1983 during the maturation of reticulocytes (20), and are significant indicators of intercellular communication within multicellular organisms (19). They play crucial roles in both normal physiological processes and in the development of diseases (21), including but not limited to material transmission, signal transduction, cell survival, apoptosis, and cell proliferation. Based on their source and dimensions, generalized EVs can be classified into three categories: Apoptotic bodies (with a diameter ranging 100 nm to 5 μm), Microvesicles (with a diameter ranging 50 nm to 1 μm), and Exosomes (with a diameter ranging 50–150 nm) (22). Apoptotic bodies emerge from the plasma membrane of apoptotic cells and typically encompass organelles and DNA fragments derived from parent cells (23). Microvesicles are extruded from the cell membrane and discharged into the extracellular milieu predominantly through a “budding” mechanism (24). Exosomes are vesicles that are secreted by the plasma membrane and undergo invagination and inward budding (25), ultimately displaying a distinctive cup-shaped morphology characterized by the expression of CD9, CD63, CD81, ALG-2-interacting protein X (ALIX), tumor susceptibility gene 101 (TSG101), heat shock cognate 71 kDa protein (Hsc70), and major histocompatibility complex (MHC) class II markers (26, 27). However, in numerous studies, no clear distinction is made between “exosomes” and “EVs,” and the two terms are often used interchangeably.

### 2.2 Composition of exosomes

The term “exosome” was coined by Johnstone et al. in 1987 (28). The exosomes were cup-shaped, with a suspension density ranged 1.10–1.21 g/ml (29). Various specific proteins such as membrane transport and fusion proteins (guanosine triphosphatase [GTPases], annexins, and flotillin) are used as markers on the surface of exosomes. Proteins (ALIX and TSG101) are involved in the biogenesis of multivesicular bodies (MVBs), four-molecule transmembrane proteins (CD9, CD63, and CD81), and heat shock proteins (Hsps, Hsp70, and Hsp90) (30–32).

Exosomes contain a diverse array of microRNAs (miRNAs), proteins, cytokines, and lipids that facilitate intercellular communication, enabling the transmission of vital biological information between neighboring and remote cells (33, 34). The lipids present in the samples consisted of cholesterol, sphingomyelin, ganglioside GM3, and externalized phosphatidylserine. The nucleic acids detected included messenger RNA (mRNAs), transfer RNA (tRNA), long non-coding RNA (lncRNA), mitochondrial DNA (mtDNA), and miRNA (35, 36).

### 2.3 Biogenesis of exosomes

**Figure 1:**
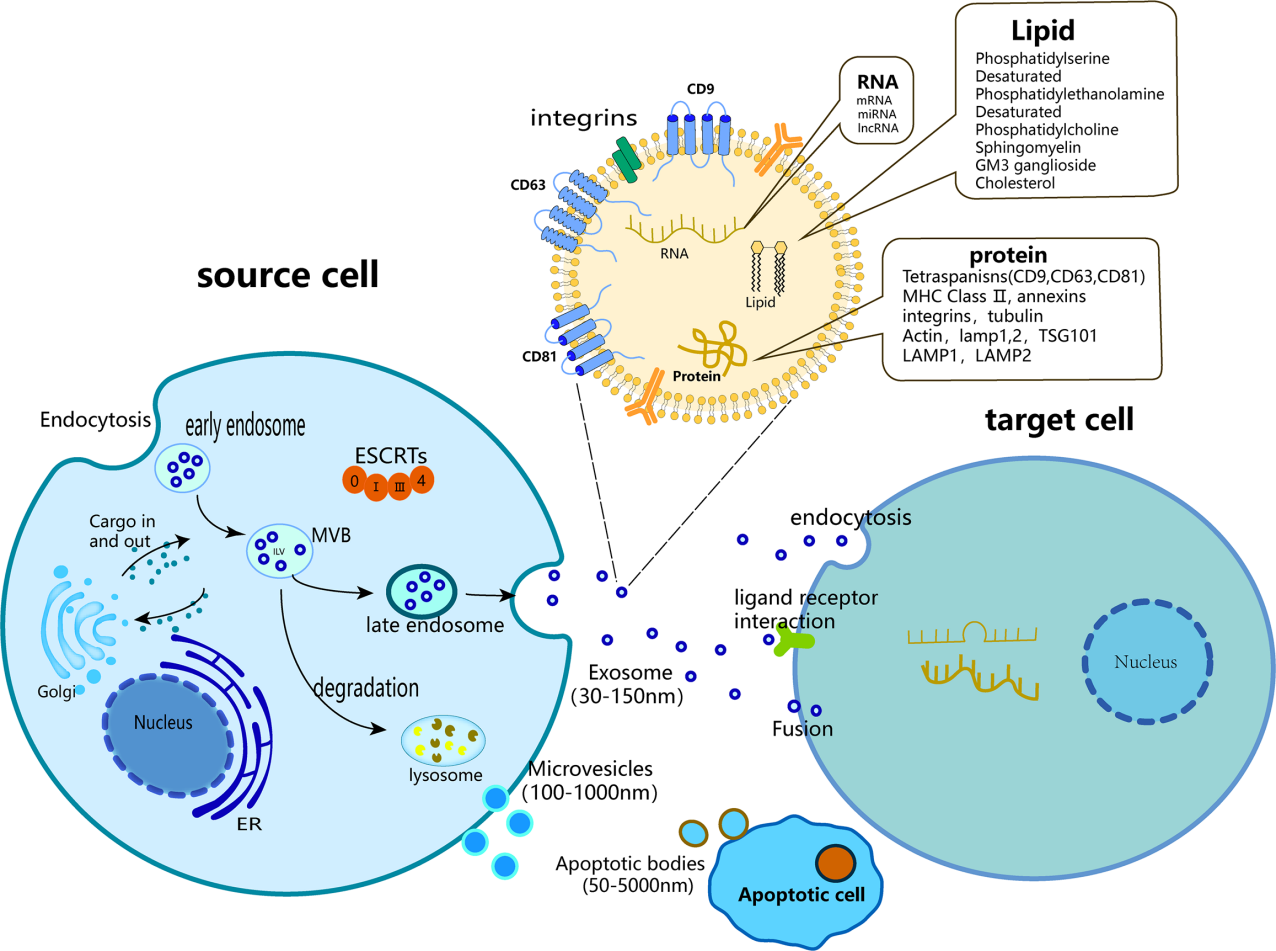
Biogenesis of three major subtypes of EVs including exosomes, microvesicles and apoptotic bodies.

The generation of exosomes consists of three main stages: (1) invagination of the plasma membrane to form endocytic vesicles and (2) inward germination of the endosomal membrane to form (MVBs) or early exosomes, leading to a gradual accumulation of luminal vesicles (ILVs) that engulf cytoplasmic components within MVBs. In addition, proteins are incorporated into invaginated membranes. During this process, proteins, mRNAs, and ncRNAs are encapsulated within luminal ILVs (37, 38). (3) In addition to some MVBS entering the lysosome and being degraded, most MVBS fuse with the plasma membrane together with early exosomes and secrete cystic contents to form exosomes (39–43). Among these, the soluble N-ethylmaleimide-sensitive factor attachment protein receptor (SNAREs) complex and endosomal sorting complex (ESCRT) are involved in exosome formation (43–45). The ESCRT consists of ALIX, ESCRT-II, charged multivesicular protein 2A (CHMP2A), CHMP4A/B/C, and vacuolar protein sorting 4 (VPS4) (46). The anterograde and retrograde protein sorting steps between the Golgi and plasma membranes are implemented by v-SNAREs and t-SNAREs (47). Three distinct mechanisms of intercellular communication are mediated by exosomes: (1) upon phagocytosis, exosomes release “messenger substances” into the cytoplasm and subsequently reform vesicles within the recipient cells; (2) following fusion with the plasma membrane, “messenger substances” are released into the cytoplasm; and (3) the ligand on the exosome binds to the receptor located on the target cell membrane (48–50). Exosomes or EVs undergo endocytosis and are internalized by recipient cells, subsequently releasing their contents through reverse fusion. The endocytic pathways available for this process are varied and include clathrin-mediated endocytosis and clathrin-independent pathways, such as caveolin-mediated uptake, macroendocytosis, phagocytosis, and lipid raft-mediated internalization (51) (**Figure 1**).

### 2.4 Functions of exosomes

Exosomes, an emerging cell-free modality for wound management, possess several noteworthy attributes including resemblance to their cellular source, prolonged efficacy, facile transportability, minimal immunogenicity, controllable concentration, and adaptability to the surrounding microenvironment (39, 52–55). Exosomes represent a novel avenue for wound healing because they possess the ability to impede inflammation, stimulate angiogenesis, and facilitate cellular proliferation and migration (56–58).

During the initial stages of exploration, exosomes were believed to be the mechanism by which cells expel waste materials. Subsequent investigations revealed that exosomes play a crucial role in sustaining regular physiological functions, such as preserving progenitor cell function (59), upholding immune tolerance within the body (60), and regulating cell apoptosis through diverse signaling pathways (61). As research has progressed, it has become evident that exosomes are implicated in nearly all pathological and disease processes. Exosomes have demonstrated significant diagnostic and therapeutic potential in various clinical conditions, with a considerable body of research concentrating on their application in tumor treatment. Tumor cells secrete a substantial number of exosomes harboring miRNAs that can serve as noninvasive tumor markers for diagnosis. For instance, miRNAs present in exosomes, such as miR-21, miR-141, and miR-200, can be used as tumor markers for the detection of ovarian cancer (62). The use of miR-125a-3p as a diagnostic indicator for the detection of early-stage colorectal cancer is also feasible (63). The identification of specific biomarkers such as epidermal growth factor receptor (EGFR), epithelial cell adhesion molecule (EpCAM), mucin1 (MUC1), glypican (GPC1), and Wnt2 within exosomes has demonstrated their potential for the diagnosis and prognosis of pancreatic ductal adenocarcinoma (64). Exosomes have demonstrated significant potential for targeted drug delivery and as gene carriers in regenerative medicine (65, 66). They possess a robust loading capacity, are capable of accommodating biological macromolecules, short peptides, and small-molecule drugs (67), and can be used as natural carriers of mRNA, miRNA, and intercellular proteins (68). The modified exosomes also delivered the let-7 miR to EGFR-expressing breast cancer cells, thereby reducing the tumor burden in mice (69). Rabies virus glycoprotein peptide (RVG)-modified exosomes can deliver small interfering RNAs (siRNAs) to the neurons of the central nervous system to knock down α-Syn siRNA in the brain, thereby delaying the characteristic pathological process of Parkinson’s disease (PD) (70).

Wound healing encompasses a sequence of molecular and cellular events including angiogenesis, proliferation, cell migration, tissue remodeling, and extracellular matrix (ECM) deposition. Exosomes facilitate this process through intricate mechanisms. Owing to the protection of their lipid layer from proteolytic enzymes, exosomes can effectively transmit signals to target cells such as vascular endothelial cells, fibroblasts, and keratinocytes (71). MSC-Exos can activate signaling pathways in target cells, including transducer and activator of transcription 3 (STAT3), AKT, Wnt/β-catenin, and extracellular regulated protein kinase (ERK), which play important roles in wound healing (72). The activation of signaling pathways has been observed to augment the expression of various cytokines in target cells, including, but not limited to, interleukin-6 (IL-6), STAT3, hepatocyte growth factor (HGF), insulin-like growth factor-1 (IGF-1), and stromal cell-derived factor-1 (SDF-1). These cytokines have been reported to facilitate processes such as angiogenesis, cell migration, cell proliferation, and re-epithelialization (73).

The biological functions of distinct MSC-Exos in wound repair are similar, owing to the presence of comparable bioactive factors. Nonetheless, their unique biological characteristics are contingent on the expression of specific molecules (74). Bone marrow MSC-derived-exosomes (BMSC-Exos) exhibit biological stability, low immunogenicity, and robust proliferation and viability after transplantation, primarily targeting the promotion of proliferation. Umbilical cord-derived MSC-derived exosomes (UCMSC-Exos) can be noninvasively isolated, possess low immunogenicity, and demonstrate strong self-renewal and proliferation capabilities. However, limitations exist in maintaining biological activity and delivering clinical treatment. Adipose-derived MSC-derived exosomes (ADSC-Exos) are an abundant source that can be obtained with minimal invasiveness without inducing pain. These exosomes possess pluripotent and plastic properties, are amenable to storage, and are stable in blood or body fluids. Their primary effect is on angiogenesis during wound healing.

## 3 Wound healing

**Figure 2:**
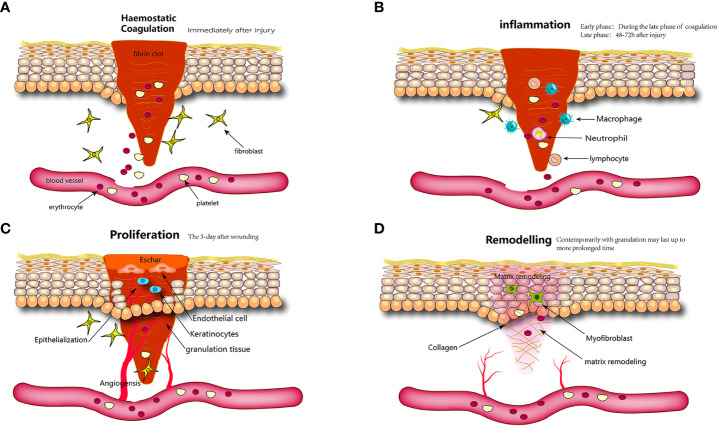
Wound healing is classically divided into four stages: **(A)** hemostasis, **(B)** inflammation, **(C)** proliferation, and **(D)** remodeling. Each stage is characterized by key molecular and cellular events and is coordinated by a host of secreted factors that are recognized and released by the cells of the wounding response.

Skin wound healing is a continuous and intertwined physiological process that is also the response of the body to injuries such as trauma, burns, or diabetic ulcers (75). It includes hemostatic, inflammatory, proliferative, and remodeling phases (76). Inaccuracies in any of these procedures may impede wound healing, resulting in the development of chronic wounds or scarring (**Figure 2**).

### 3.1 Hemostatic phase

The initial stage of wound healing is hemostasis, which commences promptly following skin trauma and involves nearly simultaneous vasoconstriction and platelet aggregation (77, 78). Concurrently, injury elicits a direct response in the form of thromboxane and prostaglandin release from the cellular membrane (79). The latter induces the contraction of vascular smooth muscle cells in the local region, resulting in a reduction in blood flow through blood vessels (76). Trauma induces pathophysiological changes that result in endothelial disruption; basement membrane exposure; and subsequent contact between flowing blood, collagen, and tissue factors, ultimately leading to thrombosis (80). Platelets are integral to this process, as they secrete various biological cytokines, including chemokines that activate immune cells to migrate toward the site of injury, and growth factors that promote the proliferation of resident fibroblasts and keratinocytes. Furthermore, platelet-derived growth factor (PdGF) stimulate the repair of vascular walls by inducing the proliferation of smooth muscle and endothelial cells. Platelet toll-like receptors (TLRs) exhibit antibacterial properties. Tissue factors function as coagulation factors that initiate a cascade of coagulation events culminating in thrombin production. Thrombin facilitates the transformation of fibrinogen into fibrin, thereby promoting the formation of durable platelet clot (81). The clot, composed of collagen and platelets, is enmeshed within cross-linked fibrin fibers, thrombospondin, and fibronectin (FN). These clots serve the dual purposes of wound coverage and hemostasis, while also serving as substrates for the migration of resident skin and immune cells (82, 83). Upon completion of its hemostatic function, the clot formed within the body undergoes resolution through a fibrinolytic system (84).

### 3.2 Inflammatory phase

The second stage of wound healing involves the initiation of the inflammatory phase, which is primarily induced by chemokines/cytokines, bacterial byproducts, and platelet-derived mediators (77). This process eradicates deteriorating bacteria, eliminate debris and damaged matrix proteins, and facilitate the creation of a wound bed that supports the growth of new tissue (85–87). Immune cells play a central role in this process by controlling bleeding and stopping infection by direct activity or by releasing a range of soluble mediators (88).

#### 3.2.1 Neutrophiles

Neutrophils represent the initial cellular responders that emerge at the site of injury and serve as the primary defense mechanism against bacterial pathogens (89). By the second day post injury, neutrophils comprise over 50% of all cells present in the wound (90). Additionally, activated neutrophils release cytokines to prolong and amplify neutrophil infiltration, thereby creating a positive feedback loop (91). Neutrophils release toxic particles, generate oxidative bursts, initiate phagocytosis, and produce neutrophil extracellular traps (NETs) to suppress infectious threats (92).

#### 3.2.2 Monocytes/Macrophages

During the initial stages of inflammation, local and systemic defense responses are triggered at the wound site. Within a timeframe of 48–96 h post-injury, monocytes originating from nearby tissues and circulating in the bloodstream are recruited to the affected area and differentiate into macrophages. These activated macrophages play a crucial role in the synthesis of nitric oxide, facilitation of angiogenesis and fibroplasia, and transition from the inflammatory phase to the proliferative phase (93). Under physiological circumstances, circulating monocytes continuously survey the endothelial wall of the vascular lumen and detect potential damage. During the inflammatory phase of wound healing, macrophages have the ability to impede infection by discharging proinflammatory cytokines, namely IL-6, tumor necrosis factor (TNF)-α, and IL-1β. Simultaneously, the initial macrophages secrete monocyte chemoattractant protein (MCP)-1 to entice additional monocytes from the bone marrow and intensify the macrophage response. Macrophages perform crucial functions during the various stages of wound healing. During the proliferative phase, macrophages facilitate the delivery of cytokines, including vascular endothelial growth factor (VEGF) and platelet-derived growth factor (PDGF), to activate endothelial cells and promote angiogenesis. Furthermore, during the remodeling phase, macrophages aid in the clearance of cell debris and excessive ECM, thereby expediting skin wound healing (94). In summary, the significance of monocytes/macrophages in wound healing has been well established. Numerous studies have shown that macrophage deficiency in mice results in delayed wound healing (95, 96).

#### 3.2.3 Lymphocytes

Lymphocytes are the cells that arrive at the site of injury. Within this population, γδ+ T cells have the capacity to generate various mediators, including IGF-1, fibroblast growth factors (FGFs), and KGFs, which facilitate the proliferation and viability of fibroblasts, immune cells, and keratinocytes. Furthermore, αβ+ T cells, a distinct subset of T cells, are also present during the inflammatory phase and contribute to the protection against pathogenic microorganisms (87, 97, 98). The inflammatory phase of wound healing involves three crucial types of inflammatory cells. However, their excessive presence can impede wound healing. Numerous studies have demonstrated that chronic wounds frequently exhibit a prolonged inflammatory phase compared to typical wounds (99–101). Subsequent investigations demonstrated that persistent inflammatory conditions impede the progression of wound repair (102). Several studies have indicated that the predominant inflammatory cells in chronic wounds are neutrophils and macrophages (103, 104).

### 3.3 Phase of proliferation

The onset of the proliferative phase commences on the third day post-injury and persists for approximately 2 weeks. During this phase, vascular endothelial cells, keratinocytes, and fibroblasts proliferate and migrate, leading to reepithelialization, angiogenesis, granulation tissue, ECM, and collagen deposition.

#### 3.3.1 Re-epithelialization

Re-epithelialization is initiated in response to the activation of inflammatory cytokines (IL-1 and TNF-), which upregulate KGF gene expression in fibroblasts shortly after skin injury. This process is dependent on the migration of epithelial cells at the wound edge and accessory structures in the dermis. The migration and proliferation of epithelial cells persist until complete coverage of the wound is achieved and an intact epithelial barrier (105). Macrophages are significant contributors to epithelialization as they regulate the proliferation and migration of keratinocytes, fibroblasts, and endothelial cells, thereby promoting tissue regeneration and the production of substantial quantities of ECM (106).

#### 3.3.2 Granulation tissue

The initial characterization and subsequent refinement of granulation tissue formation and evolution were attributed to English surgeon John Hunter and French surgeon Alexis Carrel, respectively. The ECM is composed of proteoglycans, hyaluronic acid, collagen, and elastin, which gradually form granulation tissue rather than a thrombus. Numerous cytokines and growth factors participate in this process, including the transforming growth factor-B family (TGF-b, encompassing TGF-b1, TGF-b2, and TGF-b3), IL family, and angiogenic factors. This phase can persist for several days or weeks (107). Activated fibroblasts are the primary contributors to the synthesis of a new ECM, wound contraction, and the provision of a scaffold for other cellular and component constituents, including newly synthesized ECM, neovascularization, and inflammatory cells. Ultimately, the process of wound remodeling culminates in the replacement of granulation tissue with normal connective tissue (87).

#### 3.3.3 Angiogenesis

Upon the stimulation of TNF-β, the angiogenic process focuses on the migration of endothelial cells and the formation of capillaries, as well as the accumulation of a large number of cells and a large amount of connective tissue. Neovascularization is essential for maintaining the delivery of nutrients and a physiological steady state of oxygen (87, 108). The process of angiogenesis is intricately linked to activation of microvascular endothelial cells (ECs) in the local environment. In response to hypoxia-responsive growth factors such as VEGF and PDGF, ECs in the wound environment undergo a series of cellular events, including degradation of the ECM in granulation tissue, proliferation, migration, formation of new cell-cell junctions, and branching to generate new capillaries (109). The granulation and tissue deposition require capillaries to deliver nutrients, and capillary dysfunction is likely to lead to the formation of chronic wounds.

### 3.4 Remodeling

Remodeling, also known as maturation, is the ultimate phase of wound healing and is characterized by a gradual reduction in cellular and vascular components (87). Typically, this stage commences after the culmination of granulation tissue development and persists for an extended duration, typically ranging 21 d to 1 year post-injury (110). The remodeling phase is characterized by the deposition of collagen and the gradual formation of an organized organic network. Any deviation from this process, such as excessive collagen synthesis, can have a detrimental effect on the healing strength and may result in the formation of scars or keloids (111). The remodeling phase encompasses the degradation of new blood vessels, periodic deposition of ECM, and reconstruction of granulation tissue. Vascular remodeling is a key aspect of this phase, which involves cessation of capillary growth, decreased blood flow to the wound site, and reduction in the metabolic rate at the site of injury (112–114). Ultimately, most blood vessels, fibroblasts, and inflammatory cells disappear from the wound during the remodeling phase due to cell migration, apoptosis, or other unknown mechanisms of cell death (115).

#### 3.4.1 Remodeling of the ECM

During fibrosis, myofibroblasts predominantly synthesize the ECM. These cells replace the initial fibrin clots with hyaluronic acid, FN, and proteoglycans, ultimately resulting in the formation of mature collagen fibers during the later stages of tissue repair (112, 116, 117). The ECM undergoes reorganization, degradation, and resynthesis to attain optimal tensile strength. Compared to native tissue, collagen fibers can restore approximately 80% of their initial reparative strength, with the duration of this process being a critical determinant of the ultimate strength achieved. Notably, the original strength of the tissue cannot be fully regained (113, 118).

#### 3.4.2 Granulation tissue remodeling

Wound healing involves the gradual transformation of granulation tissue into scar tissue, which is characterized by decreased cellular and vascular densities and a significant increase in collagen fiber concentration. The initial composition of granulation tissue is primarily collagen type III (COL-3); however, during the remodeling phase, a portion of the tissue is replaced by COL-1, resulting in a marked improvement in the tensile strength of the resulting scar (119). Subsequently, the concurrent processes of collagen type I and type III (COL-1 and COL-3) breakdown take place (87, 116). In subsequent phases, fibroblasts present in the granulation tissue undergo phenotypic transformation into myofibroblasts, which is accompanied by the temporary expression of smooth muscle actin (120).

## 4 Mechanisms of the effect of MSC-Exos on wound healing

Skin wound healing is a multifaceted and systematic reparative process that encompasses inflammation, cellular proliferation and migration, angiogenesis, and ECM deposition and remodeling. Numerous studies have demonstrated beneficial effects of MSC-Exos on all facets of wound healing. This review aimed to elucidate the mechanisms underlying the effects of three primary exosomes derived from MSCs on wound healing.

### 4.1 BMSC-Exos

**Figure 3:**
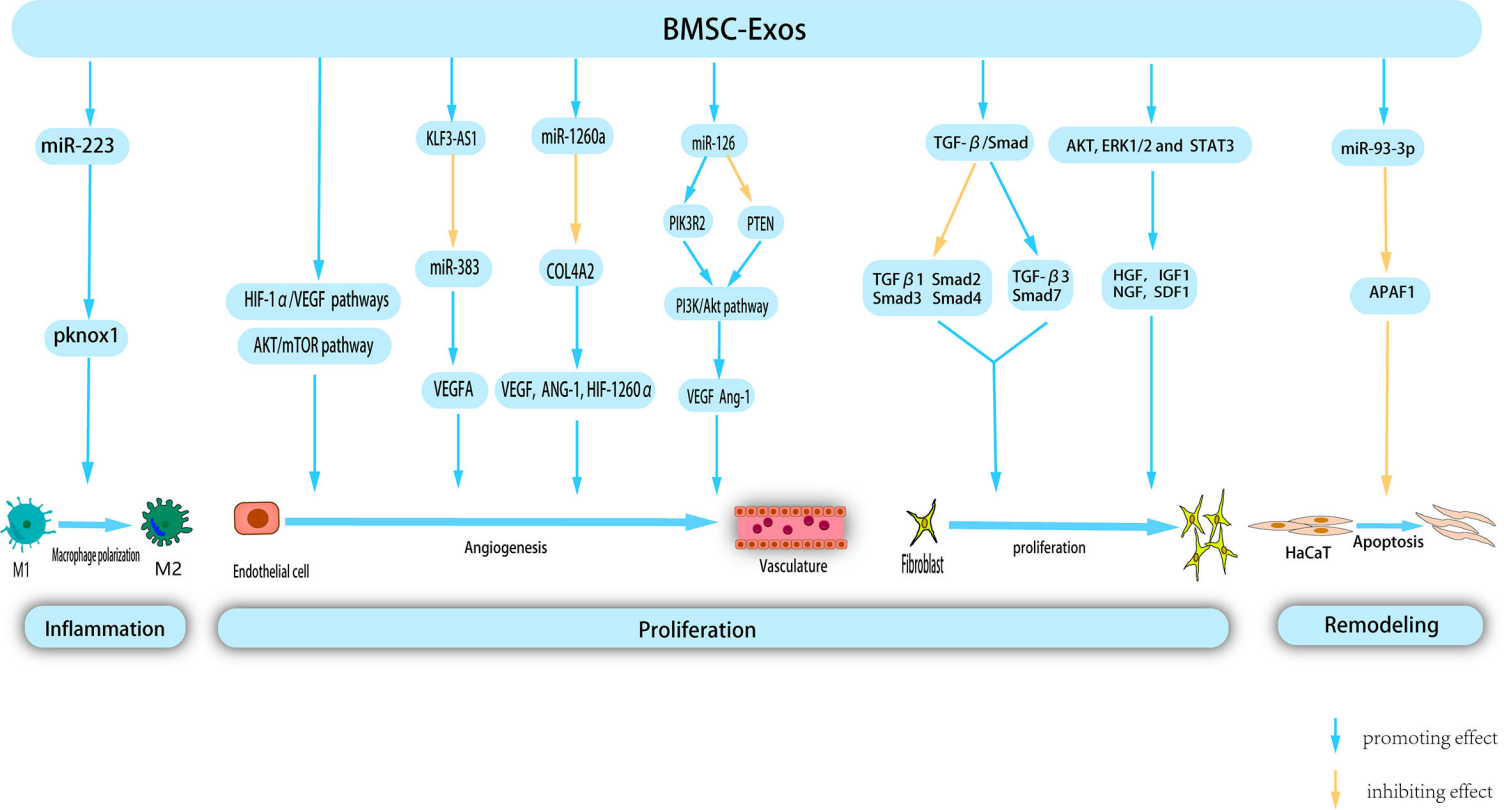
BMSC-Exos exert therapeutic effects on wounds through multiple mechanism. BMSC-Exos possess certain nucleic acids, which enable them to modulate macrophage polarization, thereby inhibiting inflammation. Additionally, during the proliferation phase, BMSC-Exos promote angiogenesis, and proliferation and migration of fibroblast. Morover, BMSC-Exos inhibit Apoptosis of HaCat in remodeling phase, thereby providing a novel approach to the treatment of cutaneous wounds.

BMSCs were the earliest isolated MSCs, and have been extensively studied and used in stem cell therapy. Recent studies have highlighted the positive effect of BMSC-Exos on all facets of wound healing, resulting in accelerated healing (**Figure 3**).

#### 4.1.1 Anti-inflammatory effect

Administration of BMSC-Exos expedites polarization of M2 macrophages during the inflammatory phase of wound healing, resulting in a reduced duration of the inflammatory process. Luo et al. conducted a study wherein they observed that intramuscular injection of BMSC-Exos following muscle contusion led to a decrease in inflammation levels. This has been attributed to the promotion of M2 macrophage polarization and anti-inflammatory factor expression, as well as a decrease in the production of inflammatory cytokines within the inflammatory microenvironment (121). Another study proposed that systemic administration of BMSC can facilitate their migration to the wound site and induce the polarization of macrophages toward the M2 phenotype, consequently enhancing the process of wound healing. Subsequent research has shown that miR-223 derived from BMSC-Exos targets pknox1 to regulate macrophage polarization (122). The etiology of chronic diabetic wound injury is primarily attributed to persistent inflammatory response, weakened angiogenesis, low immune function, and bacterial proliferation. Geng et al. developed a novel hydrogel (MSC-Exos@CEC-DCMC HG) composed of carboxyethyl chitosan (CEC) and dialdehyde carboxymethyl cellulose (DCMC) that incorporated BMSC-Exos to treat chronic diabetic wounds. A previous study revealed that BMSC-Exos facilitate the conversion of M1 to M2 macrophages, mitigate the adverse effects of inflammation, and consequently regulate the inflammatory microenvironment of the wound (123). In a study conducted by Shi et al., the group treated with BMSC-Exos exhibited a higher count of M2 macrophages and a greater presence of anti-inflammatory factors in the wound area. Conversely, the occurrence of apoptosis, M1 macrophages, and associated pro-inflammatory factors was shown to be diminished compared to the control group (124).

#### 4.1.2 Promoting angiogenesis

Under hypoxic stimulation, BMSC released a significant quantity of BMSC-EXOs. These exosomes can be internalized by venous endothelial cells, subsequently stimulating *in vitro* proliferation, migration, and tube formation (125). Hypoxia-inducible factor-1α (HIF-1α) is a key regulator of angiogenesis (126). Zhang et al. (127) confirmed that the activation of the HIF-1α/VEGF signaling pathway by BMSC-Exos promotes angiogenesis and facilitates wound healing. VEGFA, a crucial regulator of angiogenesis, plays a significant role in this process. Furthermore, lncRNAs, which do not encode proteins, but regulate cellular behavior, are essential components of BMSC-derived exosomes. Evidence has shown that KLF3-AS1, carried by BMSC-Exos, promotes VEGFA signaling and wound healing in diabetes by suppressing miR-383 (128). VEGF promotes angiogenesis. microRNA-126 (miR-126), an endothelium-specific miRNA derived from BMSC-Exos that contributes to vascular homeostasis and angiogenesis. Zhang et al. demonstrated that Exo-miR-126 facilitates angiogenesis in HUVECs by activating the PI3K/AKT signaling pathway by targeting phosphatidylinositol 3 kinase regulatory subunit 2 (PIK3R2) and upregulating the gene expression of angiogenesis-related VEGF and Ang-1. Furthermore, *in vivo* experiments confirmed that Exo-miR-126 application significantly enhanced the formation of new capillaries at the wound site and promoted wound healing (129).

BMSC-Exos have been widely shown to activate multiple signaling pathways to stimulate angiogenesis. Liang et al. demonstrated that low-dose dimethylglyoxyglycine-pretreated BMSC-Exos (DMOG-MSC-Exos) exerted advanced proangiogenic properties by activating the AKT/mTOR pathway in HUVECs (130). Ding et al. (131) demonstrated that exosomes from bone marrow MSCs pretreated with deferoxamine (DFO-Exos) significantly enhanced the angiogenic potential of human umbilical vein endothelial cells (HUVECs) by activating the PI3K/AKT signaling pathway through miR-126-mediated PTEN downregulation in diabetic rats. These findings suggest that DFO-Exos may serve as a promising therapeutic agent for promoting wound healing and angiogenesis in rats with STZ-induced diabetes. Additional research has demonstrated that pretreatment may augment the pro-angiogenic potential of BMSC-Exos. Wu et al. demonstrated that the use of magnetic nanoparticles and a static magnetic field (SMF) as a pretreatment method can augment the tissue regeneration potential of BMSC-Exos. Compared with BMSC-Exos, the administration of low-dose Fe3O4 nanoparticles in conjunction with SMF (BMSC-Fe3O4-SMF-Exos) resulted in an amplified angiogenic capacity. This effect is attributed to the presence of miR-1260a, which is highly concentrated in exosomes, and its ability to suppress collagen type IV A2 (COL-4A2) while concurrently upregulating the expression of genes associated with angiogenesis (VEGF, ANG-1, and HIF-1260α) (132).

#### 4.1.3 Promoting proliferation

BMSC-Exos can effectively stimulate the proliferation of human keratinocytes (HaCaT) and human dermal fibroblasts (HDFs) by downregulating the expression of TGFβ1, suppressor of mothers against decapentaplegic 2 (Smad2), Smad3, and Smad4 and upregulating the expression of TGF-β3 and Smad7 in the TGF-β/Smad signaling pathway. Therefore, it promoted skin wound healing (133). The role of Apoptosis Peptidase Activating Factor 1 (APAF1) is primarily associated with mitochondrial apoptosis pathway signal transduction. Overexpression of APAF1 has been observed to significantly decreased HaCaT cell viability and migration. Shen et al. demonstrated that miR-93-3p derived from BMSC-Exos can restore cellular function and inhibit apoptosis in epithelial HaCaT cells by deactivating APAF1 (134). Shabbir et al. (135)provided evidence that BMSC-Exos activate significant signaling cascades (AKT, ERK1/2, and STAT3) in recipient cells, thereby inducing the expression of growth factors, including HGF, IGF1, nerve growth factor (NGF), and SDF1, which ultimately facilitates wound healing.

These findings provide a theoretical foundation for the practical implementation of BMSC-Exos in clinical settings. It is postulated that through additional investigation of its mechanism, BMSC-Exos may emerge as a novel therapeutic intervention for wound healing.

### 4.2 UCMSC-Exos

**Figure 4:**
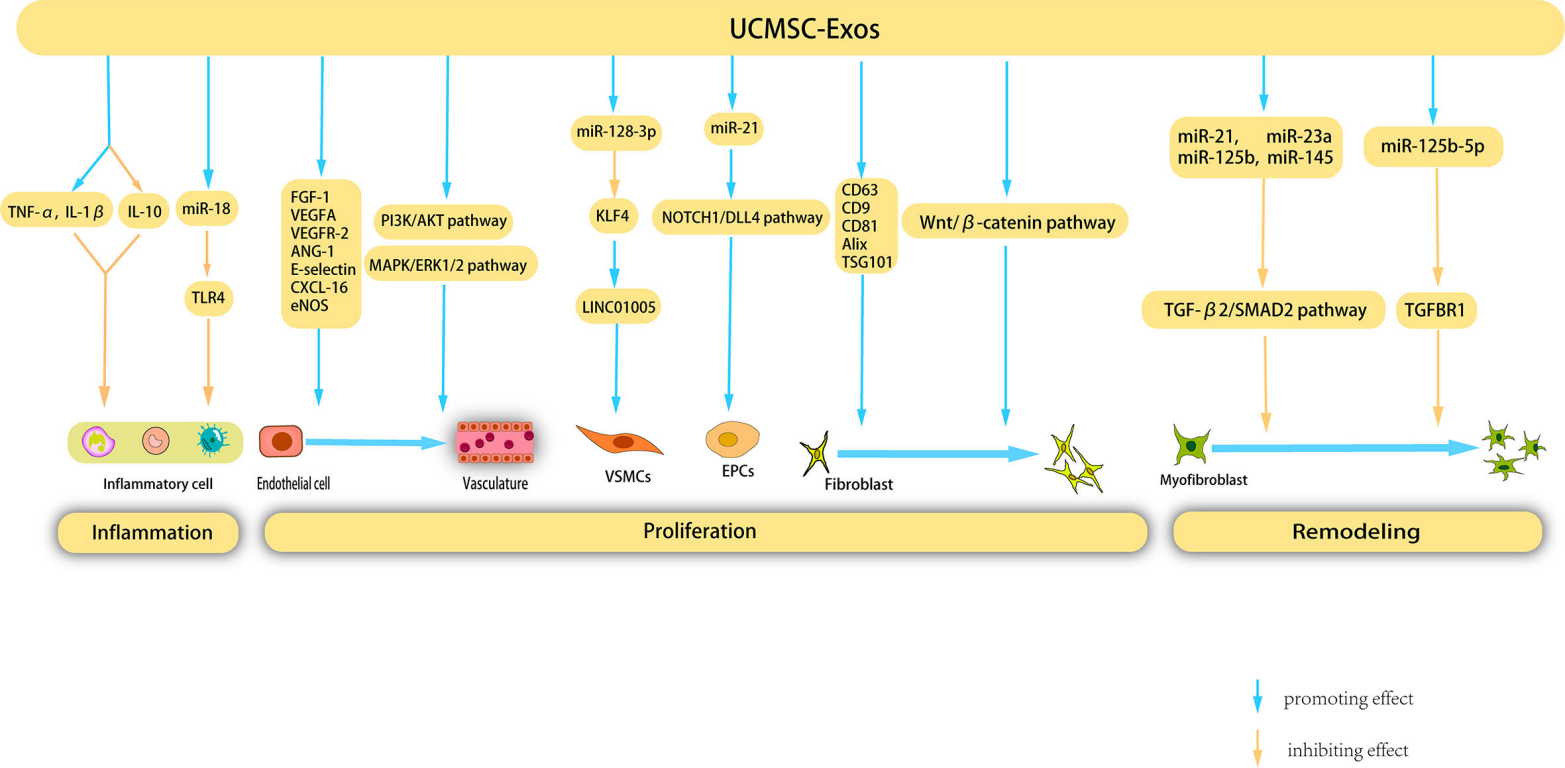
Mechanisms underlying the therapeutic effects of UCMSC-Exos on cutaneous wound healing. UCMSC-Exos contain specific cytokines and cucleic acids that facilitate the suppression of inflammatory cells, ultimately inhibiting inflammation. Furthermore, during the proliferation stage, UCMSC-Exos stimulate angiogenesis, as well as the proliferation and migration of fibroblasts, VSMCs, and EPCs. Additionally, UCMSC-Exos impede the proliferation of myofibroblast during the remodeling phase, presenting a novel therapeutic strategy for the management of cutaneous wounds.

Umbilical cord MSCs (UCMSCs) are a versatile type of stem cell that are present in the neonatal umbilical cord tissue and have significant potential for clinical applications owing to their abundant availability. Unlike other MSC-EXOs, UCMSC-Exos exhibit low immunogenicity and are devoid of tumorigenicity. Notably, UCMSC-Exos have been extensively employed in regenerative medicine and the management of diverse ailments owing to their noninvasive procurement, robust proliferative capacity, and low immunogenicity (136) (**Figure 4**).

#### 4.2.1 Anti-inflammatory effect

Previous studies have shown that UCMSC-Exos inhibit inflammation through various pathways, thereby promoting wound healing. Li et al. showed that burn significantly increased the inflammatory response of rats or macrophages exposed to lipopolysaccharide (LPS), and UCMSC-Exos reversed this response by increasing the levels of TNF-α and IL-1β and decreasing the level of IL-10. Further studies have shown that miR-181c in UCMSC-Exos reduces burn-induced inflammation by downregulating the TLR4 signaling pathway, thereby accelerating wound healing (137). Wang et al. investigated the therapeutic potential of UCMSC-Exos in the treatment of eczema using a mouse model. These findings revealed that UCMSC-Exos inhibit the proliferation of peripheral blood mononuclear cells, facilitate the differentiation of Regulatory T cells (Tregs), reduce the infiltration of lymphocytes, and significantly enhance the rate of wound closure (138).

#### 4.2.2 Angiogenesis promotion

UCMSC-Exos facilitate angiogenesis during wound healing through various mechanisms. Similar to BMSC-derived exosomes, UCMSC-Exos upregulate VEGF expression. Evidence has shown that UCMSC-Exos induce upregulation of VEGF and HIF-1α expression, thereby promoting angiogenesis in a rat model. The efficacy of HIF-1α in enhancing UCMSC-Exo-induced VEGF expression and promoting angiogenesis was confirmed using specific RNA inhibitors or siRNAs (139). Qu et al. injected UCMSC-Exos into a vein transplantation rat model and confirmed that UCMSC-Exos can accelerate re-endothelialization of the vein graft, reduce intimal hyperplasia of the vein graft, and enhance the proliferation and migration of cells *in vitro* by activating the PI3K/AKT and MAPK/ERK1/2 signaling pathways, in which VEGF plays an important role (140).

Vascular smooth muscle cells (VSMCs) are a crucial cell population involved in angiogenesis and their phenotypic modulation plays a significant role in vascular development. Exosomes derived from human umbilical vein endothelial cells (HUVECs) treated with oxidized low-density lipoprotein (ox-LDL) (ox-LDL-exo) showed high LINC01005 expression. LINC01005 functions as a sponge for miR-128-3p, leading to decrease in the expression of Kruppel-like factor 4 (KLF4). This regulatory mechanism promotes VSMC phenotype modulation, proliferation, and migration by modulating the miR-128-3p–KLF4 axis, thereby facilitating angiogenesis (141). Vascular endothelial cells are another type of cell involved in angiogenesis, and a substantial body of research has demonstrated that UCMSC-Exos possess the capacity to promote the proliferation of vascular endothelial cells. Zhang et al. (142) demonstrated that UCMSC-Exos possess the ability to enhance the proliferation and migration of vascular endothelial cells, as evidenced by cell counting kit-8 (CCK-8), scratch wound healing, transport, and tube formation assays. Li et al. corroborated that UCMSC-Exos can expedite skin wound healing in diabetic rats by stimulating endothelial cells and augmenting the expression of angiogenesis-related molecules, such as FGF-1, VEGFA, VEGFR-2, ANG-1, E-selectin, CXCL-16, and eNOS (143). Liu et al. corroborated that UCMSC-Exos expedited angiogenesis and elicited an increase in the rate of wound closure in a rat model of deep partial-thickness burns. This effect was primarily ascribed to the capacity of angiopoietin-2 (Ang-2) present in UCMSC-Exos to stimulate proliferation, migration, and tube formation of vascular endothelial cells (144). AKT, a protein kinase B, is a crucial component of several pivotal signaling pathways that facilitate cell proliferation and impede cell apoptosis. In a study by Ma et al., an adenovirus transfection system was employed to transfect AKT into UCMSC, which resulted in the acquisition of AKT-Exos. The authors confirmed that AKT-Exos augmented the proliferation, migration, and tube formation of vascular endothelial cells *in vitro* and increased angiogenesis *in vivo*. This process was significantly influenced by the high concentration of platelet-derived growth factor-D (PDGF-D) carried by AKT-Exos (145). Endothelial progenitor cells (EPCs) are precursors of vascular endothelial cells that participate in the formation of blood vessels. Zhang et al. showed that miR-21 in UCMSC-Exos serves as a potential intercellular messenger that enhances the proliferation, migration, and angiogenesis of EPCs by upregulating the NOTCH1/DLL4 pathway (146).

#### 4.2.3 Promotion of proliferation

UCMSC-Exos stimulated fibroblast proliferation and collagen synthesis through various mechanisms. Kim et al. (147) validated the ability of UCMSC-Exos to enhance HDF proliferation and collagen synthesis. Moreover, the application of UCMSC-Exos in the treatment of human skin wounds resulted in increased expression of COL-I and elastin. Another study suggested that UCMSC-Exos express typical exosome markers such as CD63, CD9, CD81, Alix, and TSG101, which significantly stimulate the migration and proliferation of mouse fibroblasts *in vitro (* 148). UCMSC-Exos stimulated HaCaT cell proliferation. Liu et al. demonstrated that UCMSC-Exos can augment the proliferation and migration of normal HaCaT cells while concurrently mitigating apoptosis and senescence in these cells. Additionally, UCMSC-Exos elevated COL-I (149). UCMSC-Exos also promoted the synthesis of ECM. Using proteomic analysis, Zhang et al. determined that 382 proteins exclusive to UCMSC-Exos participate in the composition of the ECM and extracellular structure (142). UCMSC-Exos have been observed to facilitate re-epithelialization, as evidenced by Zhang et al.’s findings of accelerated re-epithelialization in rat skin burn wounds following UCMSC-Exos treatment, along with increased *in vivo* expression of CK19, PCNA, and COL-I. The presence of Wnt4 in UCMSC-Exos has been shown to promote β-catenin nuclear translocation and activity, ultimately activating the-catenin pathway, which plays a pivotal role in wound re-epithelialization and cell proliferation (150).

The role of UCMSC-Exos in inhibiting scar formation was also demonstrated. Myofibroblast aggregation is a key factor involved in scar formation. Fang et al. (151) used high-throughput RNA sequencing and functional analysis to validate that certain miRNAs (miR-21, -23a, -125b, and -145) present in UCMSC-Exos effectively hindered myofibroblast aggregation and minimized scar formation, both *in vitro* and *in vivo*, by impeding the TGF-β2/SMAD2 pathway. Similarly, Zhang et al. (152) also confirmed that UCMSC-Exos inhibit myofibroblast differentiation. miR-21-5p and miR-125b-5p are highly enriched in UCMSC-Exos. These two miRNAs inhibit TGF-β receptor type II (TGFBR2) and TGF-β receptor type I (TGFBR1) respectively, thereby inhibiting TGF-β-induced myofibroblast differentiation in HDFS.

Although there is still a lack of high-quality evidence regarding the specific mechanism of UCMSC-Exos in wound healing, these results provide a new perspective and therapeutic strategy for the application of UCMSC-Exos in skin wound repair.

### 4.3 ADSC-Exos

**Figure 5:**
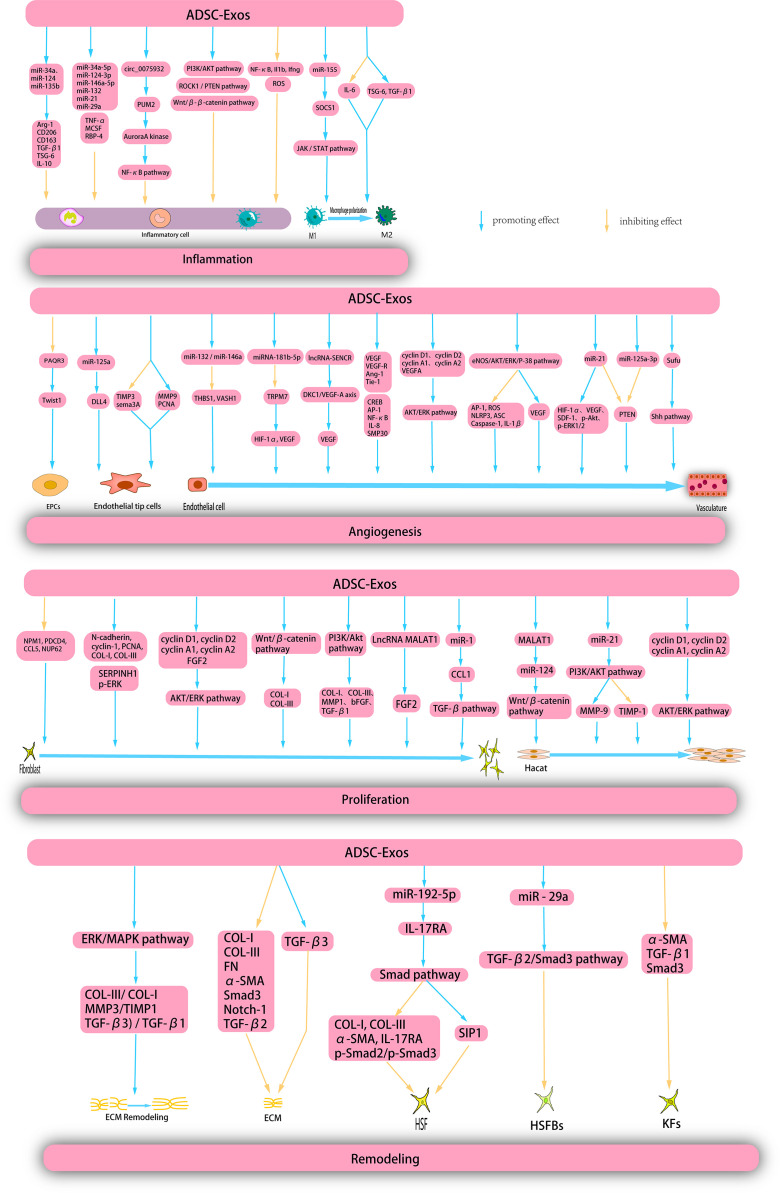
ADSC-Exos exert therapeutic effects on wounds through multiple mechanisms. ADSC-Exos exert a direct inhibitory effects on inflammatory cells and facilitate the polarization of M1 macrophages through diverse mechanisms, thereby impeding the progression of inflammation. Moreover, ADSC-Exos elicit the proliferation and migration of endothelial progenitor cells (EPCs), endothelial tip cells, and endothelial cells, thereby promoting angiogenesis. Additionally, ADSC-Exos enhance the proliferation and migration of hacat and fibroblasts during the proliferation phase. Furthermore, during the remodeling phase, ADSC-Exos facilitate extracellular matrix (ECM) remodeling, suppress the proliferation of ECM, human skin fibroblasts (HSFs), human skin fribroblasts cells (HSFBs), and keratinocytes (KFs), and inhibit scar formation.

ADSC-Exos have garnered significant attention in recent years due to their widespread distribution, ease of isolation and acquisition, and low cost, making them the most extensively researched MSC-Exos. Several studies have demonstrated that ADSC-Exos exhibit superior wound-healing properties compared to other MSC-Exos. Pelizzo et al. used a skin injury model to compare the efficacy of BMSC-Exos- and ADSC-Exos in promoting wound healing. The findings revealed that ADSC-Exos treatment exhibited a significantly superior wound healing effect compared with BMSC-Exos (153). According to transmission electron microscopy (TEM) and nanoparticle tracking analysis (NTA), ADSC-Exos were circular, cup-shaped, and uniform in size, with diameters ranging 30–150 nm. ADSC-Exos secrete numerous special protein markers, including CD63, CD81, CD9, ALIX, ANXA5, and LAMP1 (48, 55). In this section, we summarize the most recent advancements in our understanding of the mechanism of action of ADSC-Exos in the context of wound healing (**Figure 5**).

#### 4.3.1 Anti-inflammatory action

The presence of inflammation at the base level is a significant determinant of the risk of wound healing and frequently results in persistent wound-related complications. ADSC-Exos are characterized by a substantial concentration of long non-coding RNA growth arrest specific-5 (GAS5), which can effectively govern inflammatory pathways and genes in the inflammatory microenvironment that are commonly associated with chronic ailments such as diabetes and obesity, thereby facilitating wound healing (154). Furthermore, ADSC-Exos reduced inflammatory cytokine levels. Li et al. conducted a study which revealed that ADSC-Exos, which overexpress nuclear factor erythroid2-related factor2 (Nrf2), effectively decreased the levels of reactive oxygen species (ROS) and inflammatory cytokines. This intervention was shown to be effective in promoting the healing of foot wounds and reducing the ulcer area in diabetic rats (155).

Numerous studies have provided evidence that ADSC-Exos possess the ability to modulate macrophages to suppress inflammation. Specifically, ADSC-Exos may directly interact with macrophages, resulting in the inhibition of macrophage nuclear factor kappa-B (NF-κB) activity and particular pro-inflammatory genes, ultimately leading to the alleviation of macrophage-mediated inflammatory responses (156). Furthermore, some studies have confirmed that the administration of ADSC-Exos results in a reduction of the inflammatory response in mouse RAW264.7, a decrease in macrophage infiltration and pro-inflammatory cytokine levels, and an increase in the secretion of anti-inflammatory cytokines (157). ADSC-Exos can induce the polarization of M1 macrophages and expedite the inflammatory process. miR-155 produced by ADSC-Exos can be transported to macrophages, where it targets SOCS1, a confirmed miR-155 target, and modulates JAK/STAT signaling to facilitate M1 macrophage polarization and mitigate chronic inflammation (158). Heo et al. reported that ADSC-Exos down-regulate the expression of IL-6 and up-regulate the expression of tumor necrosis factor α-stimulated gene-6 (TSG-6) and TGF-β1 in an inflammatory environment, thereby promoting the polarization of M1 macrophages (159). Heo et al. revealed that ADSC-Exos secrete distinct miRNAs that play crucial roles in the anti-inflammatory processes. Specifically, ADSC-Exos exhibited high expression of miR-34a-5p, miR-124-3p, miR-146a-5p, miR-132, miR-21, and miR-29a, which have been shown to effectively mitigated inflammation and promoted wound healing (159). The following year, Heo et al. showed that ADSC-Exos can significantly up-regulate the expression of anti-inflammatory mRNA related to M2 macrophages in the inflammatory environment treated with interferon-γ and tumor necrosis factor-α. The study also showed that some miRNAs (miR-34a, miR-124, and miR-135b) were upregulated in melatonin-stimulated ADSC-Exos compared to ordinary ADSC-Exos. The up-regulation of these miRNAs contributed to gene expression of Arg-1, CD206, CD163, TGF-β1, TSG-6, and IL-10 genes, which contribute to the promotion of anti-inflammatory microenvironment (160). Blazquez et al. conducted experiments wherein they analyzed the phenotypic characteristics and functional properties of cytotoxic and helper T cells. The results indicated that ADSC-Exos exhibited an inhibitory effect on T cell differentiation and activation as well as on the proliferation of T cells and the release of IFN-γ from stimulated cells *in vitro*. Consequently, ADSC-Exos have the potential to suppress inflammation (161). Furthermore, ADSC-Exos secrete numerous immunomodulatory proteins, including TNF-α, macrophage colony-stimulating factor (MCSF), and retinol-binding protein 4 (RBP-4), which play crucial roles in the regulation of anti-inflammatory responses (162).

ADSC-Exos modulate multiple signaling pathways, resulting in the inhibition of inflammation. Heo et al. showed that ADSC-exos specifically target the ROCK1/PTEN pathway to exert their anti-inflammatory effects. ROCK1, a Rho-associated coil-containing protein kinase 1, regulates inflammation by modulating the phosphatase tensin homologue (PTEN), which in turn inhibits inflammation through the Wnt/β-catenin pathway (163). Circular RNA has_circ_0075932, is a single-exonic molecule that is expressed in normal human adipose tissue and is overexpressed in the burned skin of obese individuals. Overexpression of circ_0075932 in ADSC-Exos modulates the inflammatory response of dermal keratinocytes. This regulatory mechanism is attributed to the direct binding of circ_0075932 to the RNA-binding protein PUM2, which results in the upregulation of Aurora A kinase and subsequent activation of the NF-κB pathway (164). Wang et al. reported that hypoxia-induced adipose-derived stem cell exosomes (HypADSC-Exos) can impede the inflammatory response and enhance the healing of diabetic wounds through the PI3K/AKT signaling pathway (165).

#### 4.3.2 Angiogenesis

Numerous studies have demonstrated the ability of ADSC-Exos to facilitate angiogenesis during the repair of diabetic foot ulcers (DFUs). The presence of a high-glucose environment has been shown to impede the proliferation, migration, and angiogenesis of endothelial progenitor cells (EPCs), while increasing the expression of PAQR3 and inhibiting the expression of Twist1. Qiu et al. reported that ADSC-Exos overexpressing linc00511 can enhance angiogenesis and ameliorate DFUs by suppressing PAQR3-induced Twist1 ubiquitination and degradation and elevating Twist1 protein levels in EPCs (166). SMP30, a 34-kDa cytosolic protein commonly referred to as regucalcin, regulates intracellular Ca2+ homeostasis, ascorbate biosynthesis, and oxidative stress, and serves as a senescence marker. The absence of SMP30 exacerbates oxidative stress and impedes angiogenic activity. In patients with diabetes, the phosphorylation levels of SMP30 and VEGFR2 are significantly reduced. Li et al. demonstrated that the overexpression of Nrf2 in ADSC-Exos elevated the levels of SMP30 and VEGF and enhanced the phosphorylation of VEGFR2 to expedite angiogenesis (155).

Endothelial tip cells (ETCs) are another group of critical cells involved in angiogenesis. ADSC-Exos decreased the expression of tissue inhibitor of metalloproteinase 3 (TIMP3) and increased the expression of matrix metalloproteinase 9 (MMP9) and proliferating cell nuclear antigen (PCNA) in endothelial tip cells. Furthermore, miR-199-3p-modified ADSC-Exos targeted sema3A and downregulated it to promote proliferation and migration of endothelial tip cells (167). The enrichment of miR-125a in ADSCs-Exos results in the inhibition of Delta-like protein 4 (DLL4), an angiogenesis inhibitor, through the targeting of its 3’ untranslated region. This process ultimately promotes angiogenesis in endothelial cells by facilitating the formation of endothelial tip cells (168).

ADSC-Exos modulate angiogenic genes to facilitate angiogenesis. Previous studies indicated that miR-132 and miR-146a act as angiogenic enhancers and target the anti-angiogenic genes THBS1 and VASH1, respectively. Heo et al. showed that elevated levels of miR-132/miR-146a in ADSC-Exos regulate angiogenesis in HUVEC by suppressing the expression of antiangiogenic genes (163). Another study showed that hypoxia-treated ADSC-Exos upregulate angiogenesis-stimulating genes, deregulate angiogenesis-inhibiting genes, increase VEGF expression, and activate the protein kinase A (PKA) signaling pathway (169). VEGF expression was upregulated by ADSC-Exos. This was corroborated by Yang et al., who demonstrated that miRNA-181b-5p in ADSC-Exos targeted TRPM7 directly, leading to a decrease in TRPM7 mRNA and protein levels and an increase in the protein expression of HIF-1α and VEGF (170). Sun et al. demonstrated that ADSC-Exos elicit upregulation of enriched migration/differentiation-related long non-coding RNAs (lncRNA-sencrr) in smooth muscle and endothelial cells, activate the dyskerin pseudouridine synthase 1 (DKC1)/VEGF-A axis, and subsequently upregulate VEGF expression (171). In addition, ADSC-exos induced the activation of numerous growth factors. HypoADSC-Exos significantly increase the expression of VEGF and VEGF-R, EGF, FGF, angiopoietin-1 (Ang-1), and tyrosine kinase with immunoglobulin-like and EGF-like domains 1(Tie-1) (172).

ADSC-Exos can also stimulate angiogenesis through various signaling pathways. Evidence showed that ADSC-Exos are capable of modulating the cAMP response element binding protein (CREB), activator protein 1 (AP-1), nuclear factor-κb (NF-κB) signaling pathways, and IL-8 production in receptor HUVEC, thereby promoting angiogenesis, migration, and tube formation of HUVECs. Furthermore, proteomic analysis of ADSC-LPS-exos identified eukaryotic translation initiation factor 4E, amyloid βA4 protein, integrin β-1, and RAS-associated C3 botulinum toxin substrate 1 (RAC1) as potential candidates for enhanced exosome-mediated angiogenesis (173). This was followed by activation of the AKT and ERK signaling pathways. ADSC-Exos activated the AKT/ERK signaling pathway through the upregulation of cell proliferation markers (cyclins D1, D2, A1, and A2) and VEGFA gene expression. This phenomenon significantly enhanced the proliferation of HUVECs both *in vitro* and *in vivo* (174). Zhang et al. used an ischemic lower limb model in diabetic mice to investigate the effects of *in vivo* transplantation of ADSC-derived exosomes overexpressing glyoxalase 1 (GLO-1) (G-ADSC-Exos) on the laser Doppler perfusion index and microvessel density. A previous study revealed that G-ADSC-Exos can activate the eNOS/AKT/ERK/P-38 signaling pathway, promote VEGF secretion, and inhibit AP-1/ROS/NLRP3/ASC/Caspase-1/IL-1β secretion, thereby improving the aforementioned parameters (175). Recent studies have demonstrated that ADSC-Exos can downregulate the PTEN pathway and that miR-21 is a crucial miRNA involved in angiogenesis. An et al. used quantitative real-time reverse transcription-polymerase chain reaction (qRT-PCR) and western blotting (WB) to establish that ADSC-Exos overexpressing miR-21 up-regulated HIF-1α, VEGF, SDF-1, p-AKT, and p-ERK1/2 while down-regulating PTEN. These findings suggest that miR-21-enriched ADSC-Exos promote angiogenesis through the activation of AKT and ERK and the expression of HIF-1α and SDF-1 (176). miR-125a-3p in ADSC-Exos can also promote wound healing and angiogenesis in mice by inhibiting PTEN in the granulation tissue of wounds (177). Finally, the fusion suppressor factor (Sufu) and activation of the Sonic Hedgehog (Shh) signaling pathway were studied. Shh signalling may be a potential target in regenerative skin wound healing (178). miR-378 in ADSC-Exos can negatively regulate Sufu and activate the Shh signaling pathway, thus promoting angiogenesis (179). These results suggest that ADSC-Exos may be promising candidates for wound angiogenesis; however, further studies are needed to elucidate their angiogenic capacity.

#### 4.3.3 Promote proliferation

Numerous studies have demonstrated that ADSC-Exos facilitate fibroblast proliferation and augment collagen production. Specifically, ADSC-Exos can be internalized by fibroblasts, thereby inducing cell migration, proliferation, and collagen synthesis in a dose-dependent manner, while upregulating the expression of N-cadherin, cyclin-1, PCNA, and COL-I and -III genes. Additionally, histological analysis revealed that systemic administration of ADSC-Exos led to an increased production of COL-I and COL-III during the initial phases of wound healing (73). Choi et al. (55)demonstrated that ADSC-Exos harbor distinct miRNAs that impede the activity of proteins linked to aging and growth dysregulation, namely nuclear phosphate (NPM1), programmed cell death 4 (PDCD4), C-C motif chemokine ligand 5 (CCL5), and nucleotide 62 kDa (NUP62), thereby promoting the proliferation of dermal fibroblasts. Wang et al. injected ADSC-Exos into diabetic wounds of mice. Compared to the control group (phosphate-buffered saline [PBS] group), ADSC-Exos significantly promoted wound healing, and more collagen fibers were deposited in the late healing stage (180). ADSC-Exos stimulate fibroblasts by upregulating certain growth factors. Pi et al. reported that the long non-coding RNA metastasis-associated lung adenocarcinoma transcript 1 (MALAT1), derived from ADSC-Exos, upregulates FGF2, thereby enhancing the viability and migration of human skin fibroblasts (181). Other studies have demonstrated that the miR-19b present in ADSC-Exos stimulates collagen synthesis and fibroblast migration during wound healing. This effect is achieved by targeting CCL1 and subsequently regulating the TGF-β pathway (182).

In addition to growth factors, ADSC-Exos stimulated fibroblast formation by upregulating the expression of cell proliferation markers. A study conducted by Ren et al. revealed that ADSC-Exos induced the activation of the AKT/ERK signaling pathway through the upregulation of gene expression related to cell proliferation markers (cyclin D1, cyclin D2, cyclin A1, and cyclin A2) and FGF2. Consequently, this intervention significantly enhances the proliferation and migration of fibroblasts both *in vitro* and *in vivo (* 174). In addition, ADSC-Exos have been shown to activate associated enzymes, thereby facilitating fibroblast proliferation. Studies have reported that miRNA-146a-modified ADSC-Exos upregulate serine protease inhibitor family H member 1 (SERPINH1) and phosphorylated ERK (p-ERK), which in turn promote fibroblast migration and proliferation, ultimately leading to wound healing (183). Li et al. validated that the activation of the WNT/β-catenin signaling pathway by ADSC-Exos resulted in increased expression levels of COL-I and COL-III in fibroblasts, as well as dose-dependent stimulation of fibroblast migration and proliferation (184). Zhang et al. demonstrated that ADSC-Exos can upregulate the mRNA and protein expression of COL-I, COL-III, MMP1, bFGF, and TGF-β1 in fibroblasts by mediating the PI3K/AKT signaling pathway (185).

Numerous studies have demonstrated that ADSC-Exos possess the ability to enhance the proliferation of HaCaT cells. He et al. demonstrated that MALAT1 present in ADSC-Exos facilitates wound healing in response to H2O2 by targeting miR-124 and activating the-catenin pathway. This process promotes the proliferation and migration of HaCaT cells while simultaneously inhibiting apoptosis (186). ADSC-Exos also promote the proliferation of HaCaT cells and accelerate the re-epithelialization process by upregulating cell proliferation markers (cyclin D1, cyclin D2, cyclin A1, and cyclin A2) and stimulating the activation of the AKT/ERK signaling pathway (174). MMP have the potential to attenuate adhesion between HaCaT cells and the substrate, thereby facilitating cellular migration. Conversely, TIMP, a natural inhibitor of MMP, impedes the biological activity of MMP. Yang et al. reported that miR-21 expression is markedly elevated in ADSC-Exos, which effectively augments the proliferation and migration of HaCaT cells. This phenomenon is likely mediated by the activation of AKT phosphorylation, which, in turn, enhances MMP-9 and reduces TIMP-1 expression through the PI3K/AKT signaling pathway (187). The promotion of ECM (ECM) production. We used mass spectrometry to identify MMP6, COL-1A, CTSD, and TN-C within ADSC-Exos, which are integral to ECM homeostasis and participate in multiple signaling pathways associated with ECM repair and regeneration (188). The cytoskeletal protein vimentin plays an active role in wound healing. Parvania et al. indicated that vimentin present in ADSC-Exos facilitates fibroblast proliferation, migration, and ECM secretion, thereby reducing the duration of wound healing (189). Furthermore, pre-treatment significantly augmented the efficacy of ADSC-Exos in promoting cell proliferation. Wang et al. (165) confirmed that HypADSC-Exos significantly upregulated miR-21-3p/miR-126-5p/miR-31-5p and downregulated miR-99b/miR-146-a compared to ADSC-Exos. These miRNAs significantly promote fibroblast proliferation and migration. Further studies are required to elucidate the specific molecular mechanisms by which ADSC-Exos promote cell proliferation during wound healing and skin regeneration.

#### 4.3.4 Inhibition of scar formation

Scar formation often arises from the excessive proliferation of fibroblasts, synthesis of collagen, and deposition of ECM. However, ADSC-Exos may inhibit fibroblast proliferation, excessive collagen synthesis (73), and ECM deposition in specific cases, thereby impeding scar formation. First, miR-192-5p, which is present in ADSC-Exos, regulates the Smad pathway in hypertrophic scar fibrosis by targeting IL-17RA. This results in a decrease in the expression of key fibrotic markers such as COL-I, COL-III, α-smooth muscle actin (α-SMA), IL-17RA, and p-Smad2/p-Smad3, while increasing Smad interacting protein 1 (SIP1) expression. Consequently, miR-192-5p effectively inhibits the proliferation and migration of HSFS, collagen deposition, transdifferentiation of fibroblasts into myofibroblasts, and, ultimately, the formation of hypertrophic scars (190). Furthermore, the presence of miR-29a within ADSCs-Exos has been observed to impede fibrosis and scar hyperplasia in human hypertrophic scar fibroblasts (HSFBs) subsequent to scald injury, through the targeting of the TGF-β2/Smad3 signaling pathway, ultimately leading to a reduction in the formation of pathological scars (191). Wang et al. reported that ADSC-Exos impeded the differentiation of fibroblasts into myofibroblasts by activating the ERK/MAPK pathway. This process results in an elevated COL-3/COL-1 ratio, TGF-β3/TGF-β1 ratio, and MMP3/TIMP1 ratio, which facilitate ECM reconstruction during skin wound healing and diminish scar formation (192). Keloid fibroblasts (KFs) represent a distinctive subset of cells whose inhibition of the apoptotic program results in keloid formation. Administration of ADSC-Exos promotes KFs apoptosis and inhibits keloid formation. Research has indicated that ADSC-Exos may impede KFs proliferation and migration, while promoting apoptosis by suppressing the mRNA and protein expression of α-SMA, TGF-β1, and Smad3 (193, 194). Finally, the administration of ADSC-Exos resulted in the suppression of COL-I, COL-III, FN, α-SMA, Smad3, Notch-1, and TGF-β2 gene and protein expression in KFs, while simultaneously upregulating TGF-β3 expression. Consequently, the production of ECM is inhibited, leading to reduced keloid formation (195).

These data provided strong *in vitro* and ex vivo evidence that ADSC-Exos have potential clinical applications in wound healing. However, several unresolved issues remain regarding the practical applications of ADSC-Exos. For instance, the extant research on ADSC-Exos and wounds is predominantly short-term, necessitating further investigation of the long-term efficacy of ADSC-Exos through *in vivo* studies with large sample sizes. Furthermore, the specific therapeutic dose of ADSC-Exos that is both effective and safe remains unclear. Future research should focus on determining the biological safety range of ADSC-Exos.

## 5 Clinical applications of MSC-Exos in wound healing

As mentioned above, a large amount of evidence has shown that MSC-Exos have positive effects on skin wound healing in animal studies and preclinical tests; however, clinical studies on exosomes in skin wound healing are still insufficient. However, it is worth mentioning that some researchers have conducted systematic reviews and meta-analyses of preclinical studies of MSC-Exos, confirming their efficacy in wound healing. A systematic review of MSC-Exos by Bailey et al. suggested that administration of MSC-Exos improved diabetic wound closure compared with control, with a large observed effect (standardized mean difference [SMD] 5.48, 95% confidence interval [CI] 3.55–8.13). MSC-Exos enriched in non-coding RNAs or microRNAs further enhanced healing compared with control (SMD 9.89, 95%CI 7.32–12.46). Other outcomes, such as vessel density and number, scar width, and re-epithelialization improved with MSC-Exos administration with significant effect (196). This suggests that MSC-Exos are a promising treatment for diabetic wounds and warrants further investigation. In addition, a double-blind, randomized, first-in-human clinical trial by Kwon et al. showed that ADSC-Exos and fractional CO2 laser treatment of acne scars resulted in better improvement than the control, which broadly suggests that ADSC-Exos can provide synergy in the efficacy and safety of atrophic acne scar treatment (197). Therefore, MSC-Exos have good prospects for clinical applications.

## 6 Exploration of the combined effects of exosomes and biomaterials and Engineered exosomes

In most contemporary studies, MSC-Exos are administered through subcutaneous or intravenous injection onto the wound surface. Nonetheless, this method of delivery poses the potential hazard of sudden discharge and swift elimination due to the movement of bodily fluids. The injected exosomes exhibited a limited ability to accumulate at the intended site, resulting in a brief duration of exposure to the wound and suboptimal use of the therapeutic benefits of MSC-Exos. Researchers are exploring the potential of combining MSC-Exos with biomaterials to achieve synergistic therapeutic effects. Controlled release is a necessary attribute of biological materials, with hydrogels being the most extensively researched. Hydrogels serve as three-dimensional porous scaffolds that facilitate the accumulation of exosomes, sustain their functionality at the wound site, create a conducive milieu for cellular proliferation and ECM remodeling, enhance efficacy and safety, and synergistically expedite wound healing. Alternatively, the properties of a hydrogel allow it to fill the wound area, thereby creating an environment suitable for granulation growth (198). In general, hydrogel-based exosomes can be released continuously for a long time and exert long-lasting therapeutic effects, thereby achieving better wound healing. The use of chitosan (CS) hydrogel as a carrier for the sustained release of nanoparticles, including exosomes, has been identified as an optimal approach because of its hemostatic, antibacterial, biodegradable, and biocompatible properties. This approach ensures the sustained release of MSC-Exos, making them an ideal carrier for this purpose (199, 200). Tao et al. (201) demonstrated that the use of chitosan hydrogels loaded with MSC-Exos leads to a significant extension of exosome delivery, thereby prolonging the exposure of MSC-Exos to the wound site. Wang et al. (202)have confirmed that an exosome-loaded natural methylcellulose-chitosan hydrogel exhibited appropriate strength, a straightforward preparation process, efficient self-healing, biocompatibility, and an integrated structure that was suitable for the treatment of severe diabetic wounds. Shi et al. (203) employed a freeze-drying method to fabricate a chitosan/silk hydrogel sponge as an exosome scaffold, which was subsequently used in conjunction with MSC-Exos for wound treatment in diabetic rats. This study revealed a significant increase in the number of microvessels, thereby facilitating skin wound healing. Shafei et al. (204) designed alginate-based hydrogels loaded with MSC-Exos. The biodegradability and biocompatibility of this bioactive scaffold were evaluated, and it was demonstrated to effectively retain exosomes at the wound site in an animal model. Another investigator created a peptide-based FHE hydrogel (F127/OHA-EPL) that was injectable, self-healing, and antimicrobial, and incorporated stimuli-responsive MSC-Exos to enhance chronic wound healing and complete skin regeneration in a synergistic manner. The combination of the FHE hydrogel and exosomes demonstrated superior healing outcomes compared to exosomes alone, including enhanced wound closure rate, accelerated angiogenesis, expedited epithelialization, and increased collagen deposition at the wound site (205).

In addition to hydrogels, other biomaterials have exhibited promising outcomes in facilitating the use of exosomes for the advancement of wound healing. Graphene-Based Nanomaterials (GBNs) have emerged as a focus of biomaterial research. The substantial specific surface area and drug-loading capacity of GBNs contribute to augmentation of the efficacious dose of MSC-Exos at the wound site, whereas the robust sustained release capability of GBNs enables MSC-Exos to attain prolonged action duration, retention rate, and stability (206). The combination of exosomes and dressings increases their efficacy. Shiekh et al. (207)devised and assessed a wound dressing, OxOB, which was enriched with exosomes that released oxygen and antioxidants. This dressing exhibits remarkable potential for mitigating oxidative stress, promoting angiogenesis, and augmenting collagen remodeling, all of which are advantageous for the healing of diabetic wounds. Xiao et al. (208) have developed a scaffold dressing for a human acellular amniotic membrane (hAAM) loaded with MSC-Exos. The hAAM exhibited good swelling and moisturizing properties. A moist environment is essential for wound reepithelialization (209). The hAAM scaffold dressing was deemed highly appropriate for the delivery of exosomes. hAAM-Exos exhibit inflammatory regulatory functions, induce angiogenesis, and enhance ECM production, thereby expediting the healing of diabetic wounds. The exosome-carrier complex demonstrated superior healing outcomes compared with exosomes alone, indicating the synergistic impact of the sustained release of MSC-Exos.

**Figure 6:**
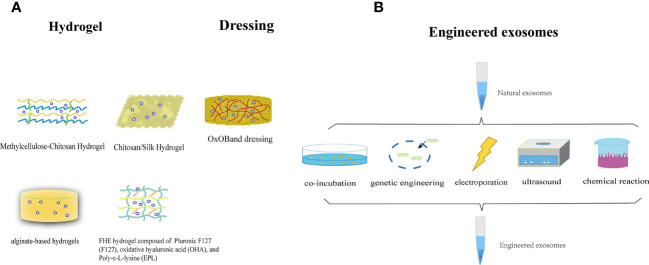
**(A)** The utilization of MSC-Exos in conjunction with biomaterials to attain a mutually reinforcing therapeutic outcome. **(B)** Illustration of the synthesis of engineered stem cell exosomes.

The efficacy of natural exosomes is hindered by several limitations including low yield, impurities, and lack of targeting (210, 211). Bioengineered exosomes may be a promising solution to this problem. There are many methods for engineering exosomes, such as modifying parental cells and directly processing stem cell-derived exosomes. These methods include co-incubation, genetic engineering, electroporation, ultrasound, parent cell surface modification, and artificial synthetic engineering. Engineered stem cell exosomes can be loaded with nucleic acids, proteins, or other small molecules (212). Engineered MSC-Exos have the potential to enhance various aspects of exosomes, including yield, purity, targeting, drug delivery, and therapeutic efficacy (213, 214). The collective use of these strategies may ultimately improve the therapeutic efficacy of exosomes for skin wound healing and regeneration (**Figure 6**).

## 7 Summary and prospects

Recently, researchers conducted in-depth studies on the role of MSC-Exos in wound repair. MSC-Exos-based therapy is emerging as a promising technology for promoting wound healing with minimal scarring. MSC-Exos represent a potential solution to the limitations of stem cell therapy, including immune rejection, ethical concerns, tumorigenicity, and malformation. Exosomes offer numerous advantages such as accessibility, ample supply, convenient storage and transport, high stability, sustained viability, small size, efficient delivery, non-proliferation, ease of quantification, and targeted recruitment to the site of injury. Furthermore, MSC-Exos contain a diverse array of proteins, lipids, and RNA molecules that confer enhanced safety and greater potential for tissue regeneration than single-cytokine therapies.

Consequently, MSC-Exos are superior to other wound therapies. In summary, despite the distinct differentiation capabilities of exosomes originating from various MSCs, they exhibit greater uniformity in fostering wound healing. Presently, most of the mechanisms elucidating the impact of MSC-Exos on wound healing have been investigated in rodents, and the extrapolation of animal physiology to humans is not direct. Therefore, it is imperative to conduct additional clinical trials using exosomes derived from human sources to establish the therapeutic potential of MSC-Exos for wound repair in a larger patient population. Furthermore, most current investigations into the mechanism of MSC-Exos rely on the *in vitro* isolation of these exosomes in a standard medium, thereby disregarding the interplay between the paracrine effect of MSC and their microenvironment. MSC-Exos produced under various microenvironmental conditions, such as hypoxia, exhibit distinct components and diverse biological effects. Most studies have failed to consider this particular aspect. Furthermore, the extraction and purification techniques used for exosomes are considerably intricate, and there is currently no universally accepted methodology appropriate for large-scale pharmaceutical production. This may impede the reproducibility of the research findings. Prompt isolation of the required quantity of exosomes to satisfy our requirements was not feasible. In addition, there is a lack of established criteria for the identification, quantity, size, purity, and content of exosome-based therapeutic agents. However, a complete understanding of the biological safety, efficacy, reproducibility, potency, formation mechanism, and biological function of exosomes in promoting wound healing remains elusive. The resolution of these issues would have a substantial impact on wound repair and skin regeneration through the use of MSC-Exos as a cell-free therapy.

## Author contributions

XQ: Conceptualization, Writing – original draft, Writing – review & editing. JH: Writing – original draft, Writing – review & editing. XW: Conceptualization, Writing – review & editing. JW: Funding acquisition, Writing – review & editing. RY: Funding acquisition, Writing – review & editing. XC: Funding acquisition, Writing – review & editing.
